# Combined SVM-CRFs for Biological Named Entity Recognition with Maximal Bidirectional Squeezing

**DOI:** 10.1371/journal.pone.0039230

**Published:** 2012-06-26

**Authors:** Fei Zhu, Bairong Shen

**Affiliations:** 1 Center for Systems Biology, Soochow University, Suzhou, Jiangsu, China; 2 School of Computer Science and Technology, Soochow University, Suzhou, Jiangsu, China; 3 Institute of Biomedical Information Engineering, Soochow University, Suzhou, Jiangsu, China; 4 Department of Bioinformatics, Medical College, Soochow University, Suzhou, Jiangsu, China; Université de Nantes, France

## Abstract

Biological named entity recognition, the identification of biological terms in text, is essential for biomedical information extraction. Machine learning-based approaches have been widely applied in this area. However, the recognition performance of current approaches could still be improved. Our novel approach is to combine support vector machines (SVMs) and conditional random fields (CRFs), which can complement and facilitate each other. During the hybrid process, we use SVM to separate biological terms from non-biological terms, before we use CRFs to determine the types of biological terms, which makes full use of the power of SVM as a binary-class classifier and the data-labeling capacity of CRFs. We then merge the results of SVM and CRFs. To remove any inconsistencies that might result from the merging, we develop a useful algorithm and apply two rules. To ensure biological terms with a maximum length are identified, we propose a maximal bidirectional squeezing approach that finds the longest term. We also add a positive gain to rare events to reinforce their probability and avoid bias. Our approach will also gradually extend the context so more contextual information can be included. We examined the performance of four approaches with GENIA corpus and JNLPBA04 data. The combination of SVM and CRFs improved performance. The macro-precision, macro-recall, and macro-F_1_ of the SVM-CRFs hybrid approach surpassed conventional SVM and CRFs. After applying the new algorithms, the macro-F1 reached 91.67% with the GENIA corpus and 84.04% with the JNLPBA04 data.

## Introduction

The development of biotechnology is contributing to the rapid growth of the biological literature. For example, PubMed (http://www.ncbi.nlm.nih.gov/pubmed/.), a free resource that is developed and maintained by National Center for Biotechnology Information (NCBI), contains more than 20 million citations of biomedical literature from MEDLINE, life science journals, and online books. The enormous volume of biological literature available provide a massive data resource for researchers, but it also a challenge for mining new information and discovering new knowledge, which has become a very important research subject.

Biological named entity recognition can be regarded as a sequence segmentation problem where each token in a sequence is assigned a biological name label (e.g. PROTEIN, DNA, RNA, CELL-LINE, CELL-TYPE,), which can be used to identify specified biological terms in text [1]–[2], or label OTHER which represents the term isn’t a predefined type of biological one. Biological named entity recognition has a key role in biological text mining. It is fundamental for biological information extraction and mining techniques [2]–[6], such as biological relation extraction [7]–[8].

However, it is difficult to correctly identify biological terms in text because they use alphabets, digits, hyphens, and other characters [6], [9]–[12]. Arbitrarily referring to biological terms makes it even harder to conduct automatic recognition using computers. In biological text, biological named entities are usually multi-word phrases and some have prefixes and/or suffixes, which makes it harder to determine the boundaries of terms. Biological terms are also affected by their context. In some cases, a biological term has different meaning among species. As a result, it is difficult for computers to recognize biological terms automatically.

Identifying biological terms from text is very important in bioinformatics. In this study, we propose a novel approach for biological named entity recognition.

### Related Work

Biological term recognition is one of the hottest research areas. Many researchers are interesting in mining biomedical terms from text, which is a key step in extracting of knowledge with an overall aim of identifying specific terms, such as genes, proteins, diseases and drugs [1]–[2].

In general, several methods are used for biological named entity recognition [9], [11], i.e., dictionary-based approaches [12], rule-based approaches, and machine learning-based approaches. However, dictionary-based approaches tend to miss undefined terms that are not mentioned in the dictionary [12]. The overall results of dictionary-based approaches rely heavily on a predefined dictionary. There is an enormous number of biological terms and new terms are constantly emerging, so it is impossible to produce a complete dictionary containing all biomedical terms. Therefore, the use of a dictionary can provide the highest precision, but we can also miss many terms. In rule-based biological term recognition systems, the rules used for identifying terms are critical, but there are generally no recognition rules that cover all cases [12]. Machine learning-based approaches train models using a training data set and the models can identify predefined types of terms.

Machine learning approaches are now a mainstream method of named entity recognition. Many algorithms are widely used, such as Bayesian approaches, Hidden Markov Model (HMM) [10], Support Vector Machines (SVM) [13]–[14], Conditional Random Fields (CRFs) [15]–[16], and Maximum Entropy (ME) [17]–[18]. For example, AbGene developed by Tanabe *et al.* [19] has an 85.7% precision rate, 66.7% recall rate, and 76.2% F1 rate when using the Bayesian method with manual post-processing. An HMM-based system designed and implemented by Zhou *et al.* [20] can recognize protein, DNA, RNA, cell-type, and cell-lines from text. Their system has a 72.55% F1 rate. Kazama *et al.* [21] used SVMs to identify protein, DNA, cell-type, cell-line, and lipid, with a 73.6% F1 rate. Tsai *et al.* [22] developed a CRF system to find protein mentions, achieving a 78.4% F1 rate. Lin *et al.* [23] used ME to recognize 23 categories of biological terms with a 72% F1 rate.

However, many methods that perform well in general text do not work as well as expected [20], [24]–[28] because there are many obstacles in biological term recognition. First, a biomedical term may have several different written forms, e.g., epilepsy and falling sickness refer to the same disease, which is a disorder of the central nervous system that is characterized by loss of consciousness and convulsions [29]. Second, an entity can be represented using different types, e.g., cancer can be used to represent a disease as well as a genus of crabs in the family Cancridae. Third, abbreviations of terms, especially arbitrarily referred abbreviations, cause even more ambiguity problems. For example, PC may refer to prostate cancer, phosphatidyl choline, or even a personal computer. Fourth, many biomedical terms are phrases or compound words, or they may have a suffix or prefix. All of these factors make it more difficult for computers to identify biomedical terms automatically [9].

Researchers have applied many methods to improve the performance of machine learning approaches, such as combining different approaches and proposing a hybrid approach, conducting post-processing after machine learning, and adding biomedical domain knowledge to machine leaning-based term identification systems. In this paper, we combined all these methods to raise the precision and recall rate.

## Results

We used SVM [6], Stanford CRFs [4] and two SVM-CRF hybrid approaches to identify biological terms from text. One SVM-CRF hybrid approach used SVM to separate biological terms from non-biological terms before using Stanford CRFs to identify the type of the biological term, while the other used SVM-CRFs to recognize biological terms before applying our proposed algorithms to improve the prediction results. The parameters for the SVM [6] and Stanford CRFs [4] used in the tests are listed in Table 1 and Table 2.

**Table 1:** Parameters for SVM in training and testing.

Parameter	Value	Parameter	Value
degree in kernel function	3	C cost of C-SVC	1
gamma in kernel function	1	tolerance of termination criterion	0.001
coef0 in kernel function	0	class weight	1

We use LIB SVM with the following settings in the experiment. The basis function is exp(-gamma*|u-v|^2^).

**Table 2:** Parameters for CRFs in training and testing.

Parameter	Value	Parameter	Value
maxLeft	1	useDisjunctive	true
useClassFeature	True	useSequences	true
useWord	True	usePrevSequences	true
useNGrams	True	useTypeSeqs	true
noMidNGrams	True	useTypeSeqs2	true
maxNGramLeng	6	useTypeySequences	true
usePrev	True	wordShape	chris2useLC
useNext	True		

We use Stanford CRFs with the following settings in the experiment.

**Figure 1:**
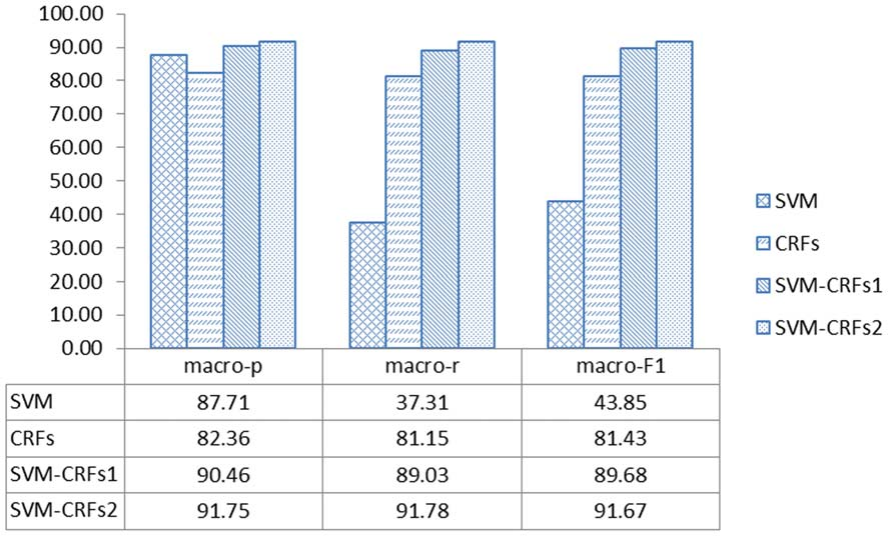
The macro-precision, macro-recall, and macro-F1 rate results using GENIA data with the four approaches. SVM-CRFs^1^ refers to SVM-CRFs without amendment while SVM-CRFs^2^ is SVM-CRFs with amendment.

In the first round, we tested four approaches using data from the GENIA corpus [5]. The F1 score for the SVM-CRFs combined approach with amendment was better than the other three approaches in five classes and it was close to the best in the remaining classes. Its macro-F1 score was greater than those of the other three approaches. The detailed testing results are shown in Table 3. The macro-precision, macro-recall, and macro-F1 rates for the results are shown in Figure 1.

**Table 3:** Testing results on GENIA data by four approaches.

	Result	SVM	CRFs	SVM-CRFs^1^	SVM-CRFs^2^
**DNA**	P	100	83.67	87.2	91.52
	R	23.39	74.57	84.83	87.43
	F1	37.91	78.86	86	89.43
**RNA**	P	100	90.87	89.93	95.02
	R	14.51	97.65	84.52	88.98
	F1	25.34	94.14	87.14	89.43
**Cell line**	P	100	82.31	91.13	93.24
	R	28.76	77.39	88.91	90.7
	F1	44.76	79.78	90.01	91.95
**Cell type**	P	35.46	79.61	91.95	93.24
	R	71	81.55	88.91	90.7
	F1	47.3	80.57	90.01	91.95
**Protein**	P	100	75.11	91.2	82.38
	R	17.35	59.19	86.99	92.92
	F1	29.57	66.2	89.04	87.33
**O**	P	90.77	82.59	91.33	95.11
	R	68.84	96.52	100	99.97
	F1	78.3	89.02	95.47	97.48

SVM-CRFs^1^ refers to the SVM-CRFs without amending and SVM-CRFs^2^ is SVM-CRFs with amending. P, R and F1 are precision, recall, and F1 respectively.

**Figure 2:**
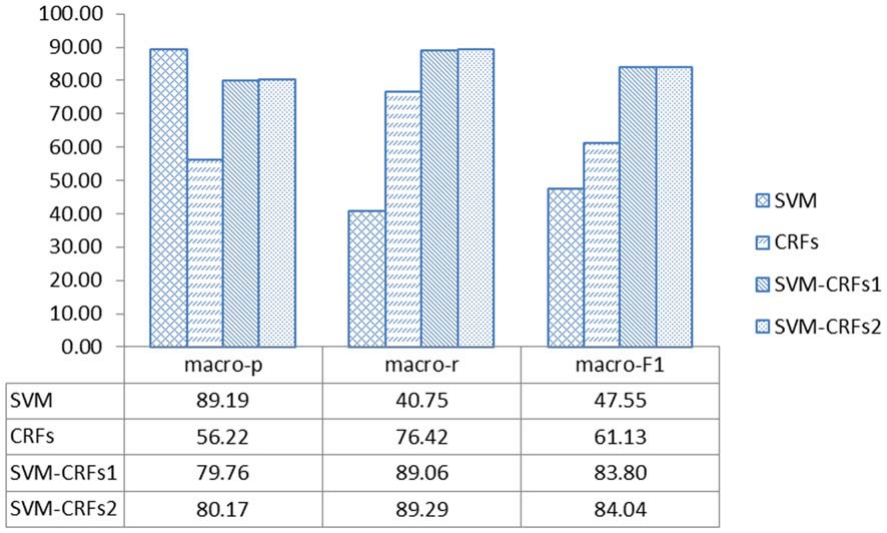
The macro-precision, macro-recall, and macro-F1 rate results using JNLPBA04 data with the four approaches. SVM-CRFs^1^ refers to SVM-CRFs without amendment while SVM-CRFs^2^ is SVM-CRFs with amendment.

In the second round, we tested four approaches using data from JNLPBA04 [7]. The F1 scores for the two SVM-CRF approaches were better than those of the other approaches. The SVM-CRFs combined approach with amendment had the highest macro-F1 score. The detailed results are shown in Table 4. The macro-precision, macro-recall, and macro-F1 rate results are shown in Figure 2.

**Table 4:** Testing results on JNLPBA04 data by four approaches.

	Result	SVM	CRFs	SVM-CRFs^1^	SVM-CRFs^2^
**DNA**	P	100	46.25	74.84	76.80
	R	27.75	92.90	87.20	87.25
	F1	43.44	61.76	81.18	81.69
**RNA**	P	100	55.84	76.66	78.32
	R	10.94	79.67	86.27	86.49
	F1	19.72	65.66	81.18	82.20
**Cell line**	P	100	53.69	76.74	79.52
	R	29.82	88.69	95.67	95.01
	F1	45.94	66.56	85.16	86.58
**Cell type**	P	42.10	52.53	79.12	81.30
	R	78.63	81.88	89.11	89.43
	F1	54.84	64.00	83.81	85.17
**Protein**	P	100	34.88	71.21	65.06
	R	24.94	69.02	89.72	91.34
	F1	39.90	46.34	79.40	75.99
**O**	P	93.01	94.15	100	100
	R	72.43	46.36	86.38	86.20
	F1	81.44	62.17	92.69	92.59

SVM- CRFs^1^ refers to the SVM-CRFs without amending and SVM-CRFs2 is SVM-CRFs with amending. P, R and F1 are precision, recall, and F_1_ respectively.

## Discussion

The results showed that the SVM-CRFs hybrid approach could identify biological terms from text well and they performed better than conventional SVM and CRFs approaches. We found in some cases, that SVM had higher precision but it tended to miss terms and unstable when trained with a small-sized data set. If the positive data are much less than the negative one, its optimal hyper plane will be biased to negative. Moreover, when the number of feature dimensions is much higher than the size of training set, over-fitting is very likely to happen. For example, monocyte macrophage lineage associated surface antigen is a protein term. However, the result by SVM is not correct
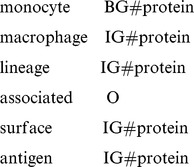
where the word associated should be tagged as IG#protein. This error is caused because the number of positive samples of the word “associated” as IG#protein is much less than that of negative ones.

The results showed that although the performance of CRFs was medium, they maintained a balance between precision and recall rate, indicating that this was a stable approach. All the results suggested that combining SVM and CRFs can provide better performance because this hybrid technique was complementary. The basic idea of our approach was to make full use of the power of SVMs as a binary-class classifier, which facilitates data labeling with CRFs. However, SVM and CRFs are the two very different algorithms, so simply combining them could cause inconsistencies. The proposed amendment algorithms were designed to correct any inconsistencies and promote their performance.

## Materials and Methods

### Materials

There are many benchmark corpuses for biological named entity recognition, such as the GENIA [5] data set, JNLPBA04 shared task data set [7], GENETAG data set [8], and MEDSTRACT data set [8]. The GENIA corpus was developed for applying natural language processing technology to biological text mining. It contains 2,000 MEDLINE abstracts with more than 400,000 words and almost 100,000 annotations of biological terms [5]. JNLPBA04 [7] has several shared tasks for natural language processing in biomedicine and its application. Bio-entity recognition is one of the tasks of JNLPBA04. The JNLPBA04 data set is often used as a benchmark data set for evaluation methods.

In the first round of testing, we divided data from the GENIA corpus into two parts, i.e., one part for training and the other for testing. We randomly picked 2000 DNA terms, 683 RNA terms, 2000 protein terms, 2000 cell line terms, 2000 cell type terms, and 2000 other types of terms for training. We then selected 400 DNA terms, 166 RNA terms, 400 protein terms, 400 cell line terms, 400 cell type terms, and 400 other types of terms for testing.

In the second round of testing, we randomly selected 2000 DNA terms, 950 RNA terms, 2000 protein terms, 2000 cell line terms, 2000 cell type terms, and 2000 other types of terms from JNLPBA04. We then picked 400 DNA terms, 118 RNA terms, 400 protein terms, 400 cell line terms, 400 cell type terms, and 400 other types of terms for testing.

### SVM Terms Identifier

SVM performs well in solving small sample size, nonlinear, and high-dimensional pattern recognition problems and other machine learning problems [30]. Assume that we are given data 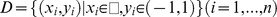 where 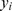 is either 1 or −1, indicating the class of 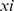. In our previous experiment [31], we used SVM to identify biological terms from text. We used word, word shape, part-of-speech, and morphology as features for identification, as shown in Table 5. The results [31] were good.

**Table 5:** Features that are generally used for SVM named entity recognition.

	Features		Features
1	All figures	12	With ‘%’
2	With figures and letters	13	With ‘,’
3	With capitalized letters	14	With ‘.’
4	All capitalized letters	15	With ‘:’
5	First letter is a capitalized letter	16	With ‘−’
6	First letter is a capitalized letter and followed by ‘.’	17	Combination of letters and ‘$’
7	With capitalized letter in the middle of the word	18	Combination of capital letters and ‘.’
8	All lower-case letters	19	Combination of letters and ‘.’
9	With two ‘/’	20	Combination of letters and ‘−’
10	With one ‘/’	21	Combination of figures, letters and ‘/’
11	With ‘$’		

SVM uses a line or surface to separate the data [30]. Thus, SVM is suitable for binary classification problems but not multiple-class problems where there are more than two candidate objective classes [32]. In most cases, name entity recognition is a multiple-class task. As a result, the initial binary SVM is not fit for most name entity recognition tasks. We can use two main types of approaches to solve multiple-class problems. One is to update an SVM kernel function that can merge the multiple classification surface problems into an optimization so as to solve multiple class classification in one pass. The alternative is to apply multiple binary classifiers until they finish the job [32].

### CRFs Terms Identifier

CRFs are often used for the labeling or parsing of sequential data, such as natural language text or biological sequences [33]. CRFs work well in named entity recognition tasks. Many features can be used in CRFs. For example, term appearance (e.g., capitalization, affixes, etc.) and orthographic features (e.g., alphanumeric characters, dashes, Roman numeral characters, etc.) are used frequently.

However, CRFs have many drawbacks. First, CRFs use a limited size of context rather than the whole text because of computational limitation, thereby limiting the contextual information. Second, splitting the context of the whole text into small pieces of context will generally separate inherent relationships among them, and simply combining these pieces of context again cannot reproduce the original context due to the loss of relationships during splitting. For example, a CRF biological term identifier uses a two-word context. The whole text could be split into many pieces of two-word contexts. As a result, the same term in the different places of the text could be tagged with different results due to the variation in the context. However, SVM deals with the whole text so it does not have such restrictions. Third, CRFs are affected by the data distribution. If we want to achieve better results, the data should have an exponential distribution. However, biological terms in texts generally do not meet this data distribution prerequisite.

### SVM-CRFs Combined Biological Name Entity Recognition

One of the new research areas in machine learning is combining useful algorithms together to provide better performance or for achieving smooth and stable performance. SVM and CRFs are two conventional algorithms that can deal with named entity recognition tasks well. As stated earlier, the feature context used by SVM is global and it does not have the same constraints as CRFs. SVM is initially the best fit for binary-class tasks and it does not perform well on multiple-class tasks. CRFs generally require more computational time and space than SVMs. Thus, although CRFs have many drawbacks, they are very good at sequential data tagging tasks, which is a typical problem in name entity recognition. Thus, we combined SVM and CRFs because they can complement and facilitate each other.

In our approach, biological named entity recognition was regarded as a two-step task. The first step was to determine whether a candidate term was a biological one. If it was a biological term, we determine its class of entity. The first step was a binary classification task where the result was either yes or no, before we could fully use SVM to complete the task. We then used CRFs to infer the type of biological term. Finally, we merged the results returned by SVM and CRFs, before performing an amendment process.

### Inconsistency Removal

In this paper, we used a BIO pattern for the resulting tags: tag that started with the character B began a term; tags starting with the character I represented the intermediate words of a term; while tags starting with the character O indicated that the word was not a biological term. For example, the tag BG#protein shows that the word is the starting word of a protein, while the tag IG#protein is an intermediate word for a protein. Thus, the following words with tags
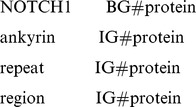
can be composed as a complete protein term: *NOTCH1 ankyrin repeat region*.

Given the statement above, we propose a phased approach (Algorithm 1) for determining whether a term is a biological term, as shown in Algorithm 1.
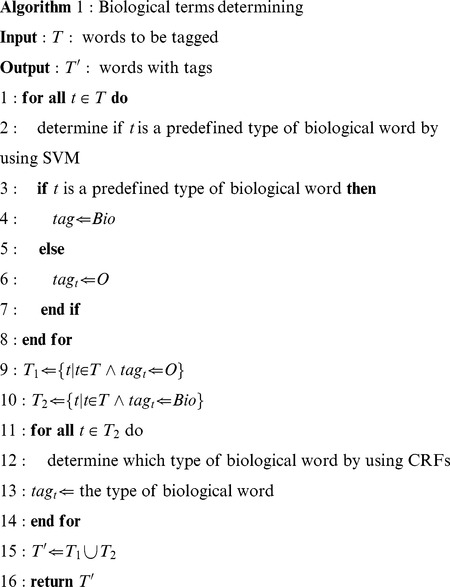



Algorithm 1 determined whether a term was a biological one. The input was the word set of all terms. The output was words with the tag *Bio* showing that the word was part of a biological term or the tag *O* showing that the word was not a biological term. Words tagged with *Bio* are further processed by CRFs to determine their biological classes.

However, SVM and CRFs are two different algorithms. Simply merging the results returned by SVM and CRFs could cause inconsistency. For example, the term *CsA treated cell* is a cell line mention. Its correct tag should be
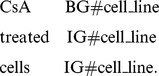



The SVM identifier predicted the word *CsA* and word *cells* as biological words, but the word *treated* was predicted as a non-biological term. The final results of the SVM and CRFs are
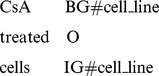



Therefore, we needed to amend any inconsistencies to improve the results. Before the amendment, we determined which terms were inconsistent. We use the following two rules to identify inconsistent terms:

- Rule 1: If the precursor and the successor of a word are both middle words of a biological term, the word should be also a middle word of the term.
- Rule 2: A term begins with a word tagged with a start tag.

Rule 1 and Rule 2 removed any inconsistencies caused by shifts in context. We used Algorithm 2 to carry out the term consistency analysis, as shown as follows.







Algorithm 2 determined word inconsistency of a term by merging the results of SVM and CRFs, and returning a pending inconsistent terms list.

### Term Length Maximizing

Using Rule 1 and Rule 2, we can identify and eliminate inconsistencies. In the example, the prediction results for the term *CsA treated cell*

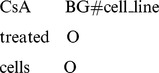
will be treated as correct, although the results are not exactly the best fit. Thus, we propose a new rule to address this type of inconsistency.

- Rule 3: The length of a biological term is expected to be as long as possible.

According to Rule 3, biological terms should be as long as possible. Using our approach, we extend a term from left to right to validate whether the extended terms are biological terms. Thus, given 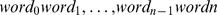, if 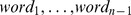 is tagged as a biological term, we have to check:
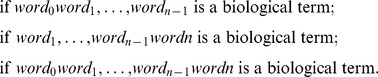



If any of the extended terms are in a biological term list, it is definitely a biological term. However, it is impossible to produce a complete biological term dictionary. Therefore, we need to make some deductions to predict the tags of the extended word.

We used a maximal forward and backward probability squeezing approach to extend the term. The maximal forward probability approach determines each forward output probability of state t on the basis of state t−1, while the maximal backward probability determines each backward output probability of state t on the basis of the state t+1 [34]. Our approach identifies the output with the maximal product result for the forward probability and the backward probability.

We assume an output sequence 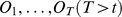
and a hidden state sequence 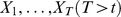. Let 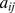 be the transfer probability from state t−1 to state t, while 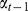 is the probability of observing all of the given data up to state t−1. At state t−1, given an output sequence 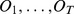 and a hidden state 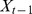, we can find the forward output using the following equations [34].
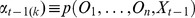
(7)

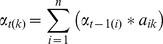
(8)


Let, 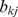 be the output probability from state t to state t +1 and 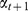 be the probability of all future data from state t +1 to state t. At state t+1, given output sequence 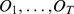 and hidden state 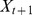, we can conduct inference and find the backward output using the following equations [34].
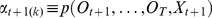
(9)

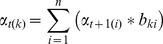
(10)


**Figure 3:**
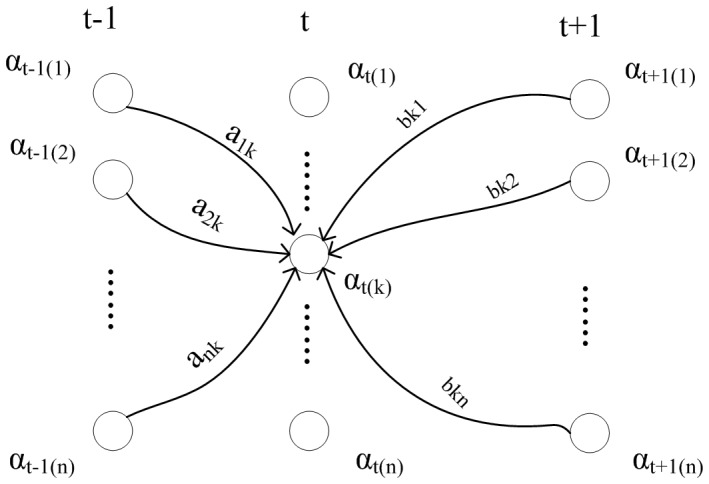
Forward and backward probability squeezing takes the product of the probability obtained by forward inference and the probability obtained by backword inference. Here 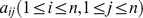 and 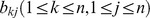 are the transfer probability, while 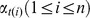 is the probability of taking 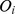.

The final result maximizes the product of the result returned by forward inference and backward inference, as shown in the following equation. An illustration of maximal forward and backward probability squeezing is shown in Figure 3.
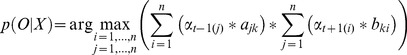
(11)


The maximal bidirectional probability squeezing method that uses the forward probability and backward probability to predict the outputs of intermediate states tends to lead to bias when dealing with states that are rare. Thus, we add positive gain to rare event cases to reinforce their probability and avoid bias, as shown in Algorithm 3.




Algorithm 3 adds positive gain to rare cases to reinforce their probability and avoid bias.

We also maintain the context window as large as possible, so the output has the maximal positive gain, as shown in Algorithm 4.




Algorithm 4 is maximal bidirectional probability squeezing, which uses the forward probability and backward probability to predict the output. Algorithm 4 also maintains a maximal context window so the output has the maximal positive gain.

When we use Rule 3 to maximize the term length, we gradually extend the context window size. We initially set the context window size for the tag 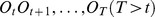
as 3. The sequence piece of the context window will then be 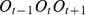, while the pending sequence is extended to 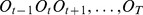. We take the piece 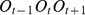
and use Algorithm 4 to infer the resulting tag 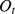. We then judge whether it is correct using Algorithm 2. If correct, the output of the sequence will be revised, but otherwise the context window will be extended left one step and right one step, making it 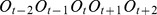. The pending sequence will also be extended to 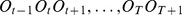. We then determine the state of 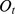 using Algorithm 4 with the context window 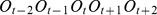. This is conducted iteratively until the predictive tag result is correct according to Algorithm 2 or we still cannot find the correct result after the whole output sequence has been treated. The amendment of the output sequence in various contexts is performed using Algorithm 5
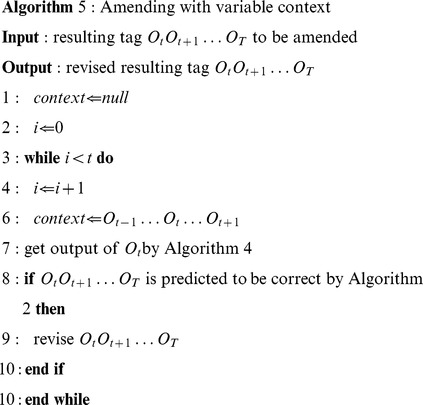



Algorithm 5 ensures that the results in context will be adaptively extended gradually.

### Performance Evaluation

We evaluate the results in terms of precision, recall rate, and F_1_ rate. Precision, recall rate, and F_1_ are given by the following equations [3].
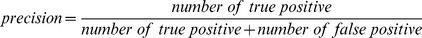
(1)

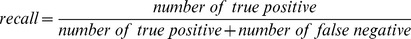
(2)

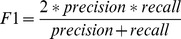
(3)


For example, when we identify a protein term, the definition of true positive, false positive, true negative, and false negative are regarded as:

True positive: protein term correctly identified as protein.

False positive: non-protein term incorrectly identified as protein.

True negative: non-protein term correctly identified as non-protein.

False negative: protein term incorrectly identified as non-protein.

We also used macro-precision, macro-recall and macro-F_1_, to evaluate the overall performance of the identifiers. Their definitions are as follows [3]:
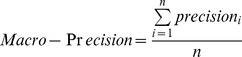
(4)

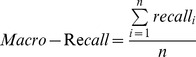
(5)

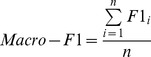
(6)


### Conclusions

The vast biological literatures provide a highly reliable information source for biological research. Mining information and finding new knowledge is a very important new subject, where the identification of biological terms is fundamental. We propose a novel machine learning approach to achieve biological named entity recognition. This approach used an SVM to determine whether the term is a biological term, before CRFs were used to infer the type of a biological term. We then judged whether the merged result was consistent in the new global context and applied an amendment approach that used maximal bidirectional squeezing with positive gain in an adaptive context algorithm for correcting inconsistent terms. The results showed that our approach could achieve biological named entity recognition and it performed better than CRFs and SVM alone.
